# Identification of biomarker panels as predictors of severity in coronary artery disease

**DOI:** 10.1111/jcmm.16244

**Published:** 2020-12-31

**Authors:** Assia Ben Braiek, Hinda Chahed, Florent Dumont, Fodha Abdelhak, Denguir Hichem, Habib Gamra, Bruno Baudin

**Affiliations:** ^1^ Service de Biochimie DMU BioGem Hôpital Saint‐Antoine, Paris Sorbonne Université Paris France; ^2^ Molecular Biology Departments Faculty of Pharmacy Monastir University Monastir Tunisia; ^3^ UMS IPSIT ‐ UFR Pharmacie Université Paris‐Saclay Chatenay‐Malabry France; ^4^ Department of Cardiology Fattouma Bourguiba Hospital Monastir Tunisia; ^5^ Department of Cardiology Farhat Hached Hospital Sousse Tunisia; ^6^ The Regional Hospital Mohamed Ben Sassi Gabes Tunisia; ^7^ INSERM UMR 1193 ‐ UFR Pharmacie Université Paris‐Saclay Châtenay‐Malabry France

**Keywords:** apolipoproteins, arterial hypertension, coronary artery disease, diabetes, matrix metalloproteinases, tissue inhibitors of matrix metalloproteinases

## Abstract

Matrix metalloproteinases (MMPs) are implicated in atherosclerotic plaque rupture and recondition. Specific tissue inhibitors (TIMPs) control MMP functions. Both MMPs and TIMPs are potential biomarkers of plaque instability. Elevated Apo‐CII and CIII and Apo‐E levels are recognized as cardiovascular disease risk factors. We aimed to establish the best blood biomarker panel to evaluate the coronary artery disease (CAD) severity. Plasma levels of MMP‐3 and MMP‐9, TIMP‐1 and TIMP‐2, Apo‐CII, Apo‐CIII and Apo‐E were measured in 472 patients with CAD evaluated by coronary angiography and electrocardiography, and in 285 healthy controls. MMP‐3 and MMP‐9 plasma levels in CAD patients were significantly increased (*P* < 0.001) compared to controls (3.54‐ and 3.81‐fold, respectively). Furthermore, these increments are modulated by CAD severity as well as for Apo‐CII and Apo‐CIII levels (*P* < 0.001). TIMPs levels were decreased in CAD versus controls (*P* < 0.001) and in inverse correlation to MMPs. Standard ROC curve approach showed the importance of panels of biomarkers, including MMP‐3, MMP‐9, TIMP‐1, TIMP‐2, Apo‐CII and Apo‐CIII, for disease aggravation diagnosis. A high area under curve (AUC) value (0.995) was reached for the association of MMP‐9, TIMP‐2 and Apo‐CIII. The unbalance between MMPs and TIMPs in vascular wall and dyslipidaemia creates favourable conditions for plaque disruption. Our study suggests that the combination of MMP‐9, TIMP‐2 and Apo‐CIII values (‘CAD aggravation panel’) characterizes the severity of CAD, that is electrophysiological state, number of involved vessels, stent disposal and type of stent.

## 1 INTRODUCTION

The main cause of coronary artery disease (CAD) is atherosclerosis, which is a multifactorial disorder identified by artery intima damage. The normal vascular intima is in balance with the synthesis and degeneration of extracellular matrix (ECM), thus any shortage in ECM synthesis causes degradation and disruption of atherosclerotic plaques, specifically the vulnerable plaque areas leading to stenosis and myocardial infarction (MI).^1^, ^2^ The major cellular and extracellular protein components of atherosclerotic plaques are apolipoproteins, fibrin(ogen)‐related antigens and matrix metalloproteinases (MMPs).^3^ MMPs, or matrixins, a class of estimated 28 endopeptidases, perform an important function in vascular inflammation and atherosclerotic plaque remodelling with potential plaque rupture via fibrous cap deterioration.^2^, ^4^ MMP‐3 is implicated in the formation of atherosclerotic plaque, has an important role in the turnover of ECM in the vascular wall leading to plaque rupture.^5^ MMP‐9 is involved in collagen degradation, vasculature remodelling, angiogenesis, inflammation and atherosclerotic plaque rupture.^5^ MMPs activities are controlled by endogenous inhibitors, that is tissue inhibitors of metalloproteinases or TIMPs.^6^ An imbalance between MMPs and TIMPs could cause large increase in MMP activity and may lead to pathological changes in the vessel wall structure associated with vascular disease, enhancing atherosclerotic plaque development and instability.^7^ Thus blood levels of MMPs and TIMPs appear as CAD risk biomarkers.^8^ We hypothesized that MMP‐3, MMP‐9, TIMP‐1 and TIMP‐2 circulating levels were irregular in patients at various severity stages of CAD. We also measured the circulating levels of apolipoproteins, namely Apo‐E, Apo‐CII and Apo‐CIII, all being biological risk factors for cardiovascular diseases and their different events, especially CAD.^9^ Apo‐CII and ‐CIII lead to hypertriglyceridemia which favours atherosclerosis development by promoting inflammation and plaque vulnerability.^10^ Apo‐E is involved in the stimulation of cholesterol efflux from macrophage foam cells in the atherosclerotic lesion, and thus in the pathogenesis of cardiovascular disease.^10^ Previous studies found that the degradation of ApoC‐II by MMPs may play an important role in the development of pathophysiological conditions with Apo‐CII deficiency such as atherosclerosis, thus MMPs are directly involved in the accumulation of cholesterol in atherosclerotic lesions^11^; also, a silenced Apo‐CIII could increase the activity of MMP‐9.^12^

We aimed to study the association between MMPs, TIMPs and apolipoproteins in Tunisian patients with CAD and established a biomarker panel able to evaluate CAD aggravation and to assess a diagnostic panel.

## 2 MATERIALS AND METHODS

### 2.1 Studied cohort

In this case‐control multicentric study, we enrolled 472 patients based on electrocardiography and coronary angiography results and 285 healthy controls who were used as a referent group for the different studied parameters. Controls and patients with CAD were matched in age and gender (no sex‐based or race/ethnicity‐based differences were present).

We eventually included 472 consecutive CAD patients undergoing coronary angiography (angiographically defined MI) either in emergency with characteristic symptoms (typical prolonged chest pain), electrocardiographic changes or worsening symptoms during hospitalization in a period of two years from December 2014 to December 2016, from 3 hospitals: in the Cardiology department of Fattouma Bourguiba hospital in Monastir, Farhat Hached hospital in Sousse, and the regional hospital Mohamed Ben Sassi in Gabes, all are in Tunisia.

We collected the clinical data in a database, including a detailed medical history, sex, age, gender, weight and height (BMI), smoking, drug abuse and alcohol intake, the presence or absence of high blood pressure (HBP), as systolic blood pressure >140 mm Hg and/or diastolic blood pressure >90 mm Hg or treated with antihypertensive treatment, the presence or absence of diabetes mellitus or hyperlipidemia, family history of CAD and significant increase in biochemical markers of myocardial injury.

The MI diagnosis was formulated independently by the cardiologists depending on the following criteria: clinical symptoms, laboratory workup, electrocardiogram and a confirmed coronary angiography (a minimum of 50% stenosis in one major coronary vessel). The clinical severity classification has to be distinguished from angiographic complexity scores (atherosclerotic burden). The case group was divided into 3 different subgroups according to the degree of complication: (a) patients with an ST‐segment elevation MI (STEMI; n = 162), the most serious cardiovascular complication, that requires urgent medical care, (b) patients without ST‐segment elevation acute MI (NSTEMI; n = 242), patients who also have a severe presentation requiring prompt medical care and (c) patients with stable angina (n = 67) being the most benign clinical presentation, but all explored by a coronary angiography. Exclusion criteria for the patient group were the presence of non‐atherosclerotic vascular disease, cardiomyopathy, valvular disease, congenital heart disease, hepatic, renal and thyroid disease, autoimmune and infectious disease, and cancer. Patients under treatment with anti‐inflammatory drugs or with any surgical proceeding in the previous 6 months were not included in this study. The medical treatment used for patients is RAASI (renin‐angiotensin‐aldosterone system inhibitors) either an ACE inhibitor or an angiotensin receptor blocker. Diabetes was treated with insulin or oral antidiabetic drugs. Lipid‐lowering agents (statin/fibrate) were introduced just after CAD diagnosis using coronarography (1‐7 days later).

285 volunteers included in the control group with no coronary arteriography abnormality and no cardiovascular disease (CVD) history, no echocardiographic regional wall motion abnormalities, no MI electrocardiographic signs, no indicative symptoms of CAD and no significant valvular abnormalities in echocardiograms. Subjects presenting any history of heart disease, severe illness limiting life expectancy, cardiomyopathy, or neoplasm, or refusal to consent were excluded.

The clinical research ethics committee of CHU Farhat Hached, Sousse approved the study protocol and total of procedures followed was in conformity with conventional guidelines (ethics committee for human research and conform to the 1975 Helsinki declaration ethical guidelines), and to participate in the study, the patients provided a written informed consent.

### 2.2 Coronary angiography examination

In order to document the coronary stenosis, all patients underwent coronary angiography. Angiocoronarographic exams were performed and interpreted using Siemens ARTIS ZEE catheter laboratory and its dedicated QCA analysis software; they were routinely executed in at least two perpendicular projections, and follow‐up angiography was performed using the same projections. The minimal luminal, per cent of stenosis diameter and lesion length were obtained. The presence of ≥50% stenosis in at least one coronary artery defines the CAD diagnosis. The coronary atherosclerosis burden was assessed using the number of stenosed coronary vessels (one, two or three‐vessel disease) as well as the severity of the proximal lesions and left main coronary artery stenosis. Moreover, the absence (stent‐) or presence (stent+) of stent implantation was recorded as well as its type, that is bare‐metal (BMS) or drug‐eluting (DES) stent. But, in our study the stent choice is irrelevant because it was never based on clear practice guidelines.

### 2.3 Biochemical analysis

Venous blood samples were collected just before coronarography, while respecting 12‐hour fasting, from all participants and collected into 5‐mL tubes containing EDTA, Na‐heparinate and no anticoagulant serum in function of dosed parameters. Serum and plasma obtained after centrifugation (3400 g, 4°C) were stored until assay at −80°C.

MMPs and TIMPs were measured in plasma heparin samples with ELISAs using the Human MMP‐9 kit from Boster Biological Technology or the Human MMP‐3, TIMP‐1 and TIMP‐2 kits from Clinisciences following the manufacturer's instructions, all including calibrators and quality controls. Each kit contains specific MMPs or TIMPs antibodies covalently bound to sets of microspheres, each having a unique spectral signature. Apo‐E, Apo‐CII and Apo‐CIII were quantified with immunoturbidimetric methods in plasma heparin samples on Konelab 20XT from Thermofisher using the corresponding kits from Kamiya Biomedical Company including calibrators and quality controls. The samples were measured in duplicates and they were diluted or not according to manufacturer's instructions. The inter‐assay and intra‐assay coefficients of variation were less than 6%.

### 2.4 Statistical analysis

Statistical analyses were performed using R software [R Core Team (2018). R: A language and environment for statistical computing. R Foundation for Statistical Computing, Vienna, Austria. URL https://www.R‐project.org/.]

Two groups of categorical variables were elucidated as numbers and for the comparison between groups; a chi‐square test and corresponding percentages were used. Two group continuous variables were demonstrated as mean ± standard deviation (SD) and Mann‐Whitney *U* tests were applied for comparison.

For continuous variable with groups more than 2 as subgroups (severity of CAD, number of stenosed vessels, type of stents), we applied a one‐way analysis of variance and made pairwise Tukey's post hoc tests between groups.

For heatmap, both dendrograms (variables and samples) were constructed using Pearson correlation’s dissimilarity and complete linkage.

Receiver operating characteristic (ROC) curves were selected from a homemade analysis workflow in order to find the best logistical regression model. Details are described below.

## 3 RESULTS

### 3.1 Baseline characteristics and classical risk factors for cardiovascular diseases

The totality of 472 CAD subjects (mean age, 57.1 ± 11.4 years; 67% male) and 285 controls (mean age, 55.01 ± 13.82 years; 65.6% male) matched in terms of age and gender were enrolled in this study. The patient group included 34.5% STEMI, 51.3% NSTEMI and 14.2% stable angina subjects. After coronary angiography examination, depending on the number of touched vessel, patients with CAD manifested either one (33.5%), two (27.8%) or three (38.8%) vessel disease. Regarding the therapeutic procedures, a total of 42.4% of patients were treated with a bare‐metal stent (BMS), 23.7% with a drug‐eluting stent (DES), and for 33.9% of patients no stenting was performed. Compared to the control group, patients with CAD showed a significant increase in the percentage of peripheral artery disease history (*P* < 0.001), a family history of CAD (*P* < 0.001), diabetes mellitus (*P* < 0.001), hypertension (*P* < 0.001) and had more elevated BMI (*P* < 0.001). In addition, statistically significant discrepancies were shown in CAD patients when compared to controls in terms of higher consumption of alcohol and tobacco (*P* < 0.001) (Table 1). Table 2 shows higher statistically significant plasma levels for most of the biochemical (lipids) parameters in CAD patients compared to controls (*P* < 0.001) (Table 2). Concerning the patient treatments, Table 3 shows no statistical variation between CAD patient subgroups when comparing the percentage of different subgroups CAD patients (STEMI, NSTEMI, stable angina) under different medical treatments; so treatment had no influence.

**TABLE 1:** Baseline characteristics of patients and controls. Values are mean ± SD for age and weight

Variable	Subgroup	Controls (N = 185)	CAD subjects (N = 472)	*P* value
Gender	Male	187 (65.6%)	316 (66.9%)	N/S^a^
	Female	98 (34.4%)	155 (32.8%)	N/S^a^
Age (y)	NA	55.01 ± 13.82	57.06 ± 11.36	N/S^a^
Cardiovascular history	With	45 (15.8%)	341(72.2%)	<.0001^a^
	Without	240 (84.2%)	131 (27.8%)	
OALL	With	3 (1.1%)	232 (49.2%)	<.0001^a^
	Without	282 (98.9%)	240 (50.8%)	
Diabetes status	Diabetic	31 (10.87%)	299 (63.34%)	<.0001^a^
	Non‐diabetic	254 (89.12%)	173 (36.65%)	
High blood pressure	HBP	36 (12.6%)	282 (59.74%)	<.0001^a^
	Non‐HBP	249 (87.4%)	190 (40.3%)	
Smoking status	Smoker	138 (48.4%)	316 (66.9%)	<.0001^a^
	Non‐smoker	147 (51.6%)	156 (33.1%)	
Alcohol status	Alcoholic	80 (28.1%)	229 (48.5%)	<.0001^a^
	Non‐alcoholic	205 (71.9%)	243 (51.5%)	
Weight (kg)	NA	71.36 ± 8.48	79.64 ± 15.17	<.0001^a^
Body mass index	<25	68 (23.9%)	122 (25.8%)	<.0001^a^
	25‐30	198 (69.5%)	182 (38.6%)	
	>30	19 (6.7%)	168 (35.6%)	

Abbreviations: N/S, not significant; NA: not applicable; OALL, obliterated arteriopathy of lower limbs.

^a^ Derived from Chi‐square test.

**TABLE 2:** Biochemical measurements in CAD patients and controls. Mean value ± SD was given for each variable

Variable	Controls (N = 285)	CAD patients (N = 472)	*P* value
Glucose (mmol/L)	4.88 ± 0.74	9.45 ± 4.94	<.0001^b^
Triglyceride (mmol/L)	1.22 ± 0.43	2.29 ± 1.38	<.0001^b^
Total cholesterol (mmol/L)	4.45 ± 0.85	5.54 ± 1.57	<.0001^b^
LDL/HDL‐chol (mmol/L)	2.80 ± 0.81	2.44 ± 1.45	0.012^b^
Apo‐CII (mg/dL)	3.42 ± 1.70	6.02 ± 4.30	<.0001^b^
Apo‐CIII (mg/dL)	10.75 ± 6.10	19.01 ± 10.91	<.0001^b^
Apo‐E (mg/dL)	6.47 ± 4.66	9.56 ± 12.18	<.0001^b^
MMP‐3 (ng/mL)	77.39 ± 53.49	274.32 ± 151.29	<.0001^b^
MMP‐9 (ng/mL)	83.44 ± 59.15	318.10 ± 159.87	<.0001^b^
TIMP‐1 (ng/mL)	327.44 ± 88.67	146.89 ± 73.12	<.0001^b^
TIMP‐2 (ng/mL)	278.85 ± 63.72	107.84 ± 45.07	<.0001^b^

^b^ Derived from Mann‐Whitney *U* tests.

**TABLE 3:** different treatment percentage (%) of CAD patient subgroups

Treatment in percentage (%)	Controls	Patients			*P* value
		STEMI (n = 162)	NSTEMI (n = 242)	Stable angina (n = 67)	
RAASI (%)	‐	79	118	35	N/S^a^
Insulin (%)	‐	70	104	29	N/S^a^
Lipid‐lowering agents (%)	‐	87	133	36	N/S^a^

Abbreviations: N/S, not significant; RAASI, renin‐angiotensin‐aldosterone system inhibitor.

^a^ Derived from Chi‐square test.

### 3.2 Baseline levels of biomarkers (MMPs, TIMPs and Apolipoproteins)

CAD patients show statistical significant increases of MMP‐3 and MMP‐9 compared to controls (3.5 and 3.8‐fold respectively, *P* < 0.001) whereas plasma levels of both TIMP‐1 and TIMP‐2 were significantly decreased in CAD patients compared to controls (2.22 and 2.28 fold respectively, *P* < 0.001). Apo‐E, Apo‐CII and Apo‐CIII plasma levels were significantly increased in CAD patients compared to controls (1.48, 1.76, 1.77‐fold respectively, *P* < 0.001; Table 2).

Comparing diabetics (N = 330) and non‐diabetics (N = 427) in all the population including 757 individuals (patients and healthy controls), there were increased levels of MMP‐3,‐9 and Apo‐CII,‐CIII (*P* < 0.001) in diabetic individuals and decreased levels of TIMP‐1 and ‐2 (*P* < 0.001; Table 4), as well as when comparing hypertensive (N = 318) to non‐hypertensive individuals (N = 439) with increased levels of MMP‐3, ‐9 and Apo‐CIII (*P* < 0.001) in hypertensive individuals and, in parallel, decreased levels of TIMP‐1 and ‐2 (*P* < 0.001; Table 5).

**TABLE 4:** Plasma values of MMPs, TIMPs and apolipoproteins according to diabetes status. Mean values ± SD were calculated from the whole cohort including CAD and control subjects

Variable	Non‐diabetic (N = 427)	Diabetic (N = 330)	P * value
Apo‐CII (mg/dL)	4.66 ± 3.63	5.54 ± 3.88	<.001^b^
Apo‐CIII (mg/dL)	14.28 ± 10.29	17.99 ± 9.71	<.001^b^
Apo‐E (mg/dL)	7.65 ± 9.75	9.37 ± 10.57	<.01^b^
MMP‐3 (ng/mL)	145.2 ± 140.5	271.3 ± 147.1	<.001^b^
MMP‐9 (ng/mL)	163.8 ± 153.4	315.2 ± 161.0	<.001^b^
TIMP‐1 (ng/mL)	259.9 ± 122.5	156.6 ± 81.10	<.001^b^
TIMP‐2 (ng/mL)	210.1 ± 103.7	123.2 ± 63.40	<.001^b^

^b^ Derived from Mann‐Whitney *U* tests.

**TABLE 5:** Plasma values of MMPs, TIMPs and apolipoproteins according to arterial hypertension status. Mean values ± SD were calculated from the whole cohort including CAD and control subjects

Variable	Non‐hypertensive (N = 439)	Hypertensive (N = 318)	*P* value
Apo‐CII (mg/dL)	4.71 ± 3.65	5.51 ± 3.88	<.001^b^
Apo‐CIII (mg/dL)	14.31 ± 9.82	18.08 ± 10.34	<.001^b^
Apo‐E (mg/dL)	8.12 ± 10.45	8.78 ± 9.71	<.01^b^
MMP‐3 (ng/mL)	163.8 ± 151.1	250.5 ± 149.6	<.001^b^
MMP‐9 (ng/mL)	186.2 ± 169.2	289.9 ± 161.8	<.001^b^
TIMP‐1 (ng/mL)	251.2 ± 124.2	164.8 ± 87.3	<.001^b^
TIMP‐2 (ng/mL)	205.1 ± 103.5	126.9 ± 68.52	<.001^b^

^b^ Derived from Mann‐Whitney *U* tests.

### 3.3 Association of different biomarkers with CAD severity, number of touched vessel and stent treatment

**FIGURE 1:**
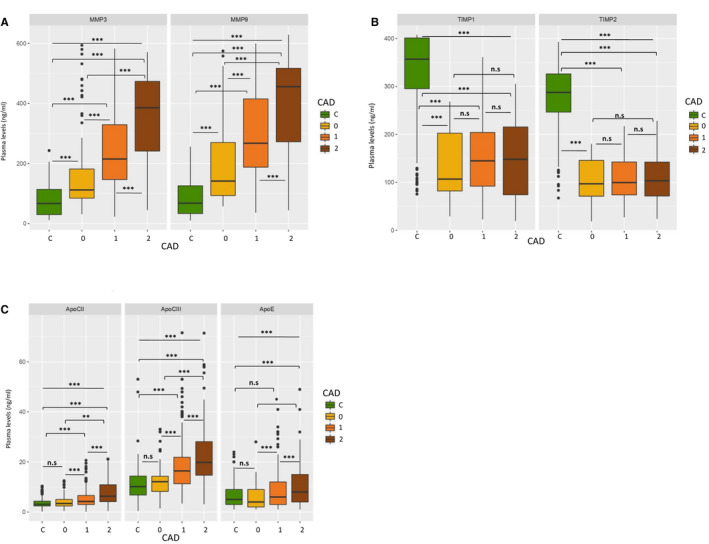
A, MMPs levels variation between CAD patient subgroups and controls. C: Controls, 0: Stable angina, 1: NSTEMI (CAD ST‐), 2: STEMI (CAD ST+), n.s: non‐significant, *P < .01, **P < .001, ***P < .0001. B, TIMPs levels variation between CAD patient subgroups and controls. C: Controls, 0: stable angina, 1: NSTEMI (CAD ST−), 2: STEMI (CAD ST+), n. s: non‐significant, *P < .01, **P < .001, ***P < .0001. C, Apolipoproteins levels variation between CAD patient subgroups and controls. C: Controls, 0: stable angina, 1: NSTEMI (CAD ST‐), 2: STEMI (CAD ST+), n. s: non‐significant, *P < .01, **P < .001, ***P < .0001. CAD, coronary artery diseases

MMP‐3 and ‐9 have higher plasma levels in patient subgroups than in the control group, the highest MMP‐3 and‐9 levels were obtained in the most severe presentation of CAD (STEMI) compared to NSTEMI and stable angina patients (*P* < 0.0001, for both). Inversely, TIMP‐1 and TIMP‐2 levels were increased in controls compared to the three CAD subgroups of severity: controls vs stable angina (*P* < 0.0001), controls vs NSTEMI (*P* < 0.0001), controls vs STEMI (*P* < 0.0001), But the level variation TIMPs have no statistically significant between the CAD subgroups. Apolipoproteins have higher significant levels in different subgroups of CAD than in the control group, the highest *P* value was between STEMI patients and controls (*P* < 0.0001, for Apo‐CII, ‐CIII and ‐E). Apo‐CII, Apo‐CIII plasma levels were significantly increased in NSTEMI patients compared to controls (*P* < 0.0001); however, no statistically significant difference was observed between controls and stable angina subgroup. Furthermore, Apolipoproteins were statistically higher in STEMI patients when compared to NSTEMI and stable angina subgroups (*P* < 0.001). Similarly, apolipoproteins were higher in NSTEMI patients compared to stable angina patients (*P* < 0.001; Figure 1A–C).

**FIGURE 2:**
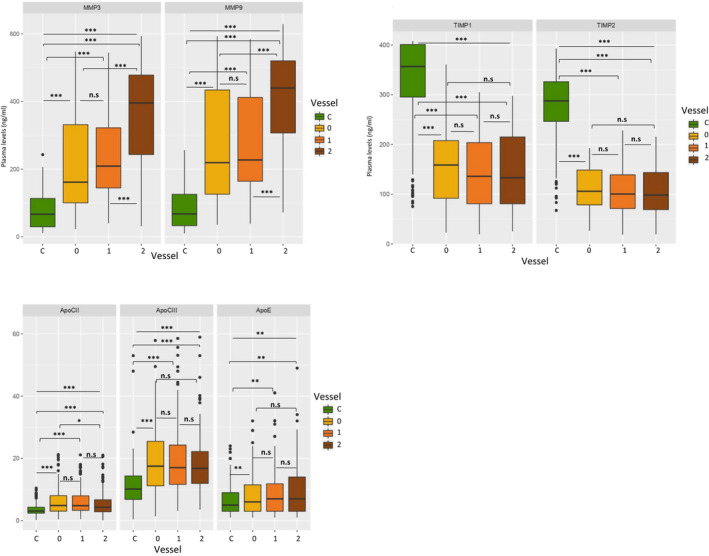
A, MMPs levels variation between different number of stenosed vessels in patient subgroups and controls. Number of stenosed Vessels, C: Controls, 0: one stenosed vessel, 1: two stenosed vessels, 2: Three stenosed vessels, n.s: non‐significant, *P < .01, **P < .001, ***P < .0001. B, TIMPs levels variation between different number of stenosed vessels in patient subgroups and controls. Number of stenosed Vessels, C: Controls, 0: one stenosed vessel, 1: two stenosed vessels, 2: three stenosed vessels, n.s: non‐significant, *P < .01, **P < .001, ***P < .0001. C, Apolipoproteins levels variation between different number of stenosed vessels in patient subgroups and controls. Number of stenosed Vessels, C: Controls, 0: one stenosed vessel, 1: two stenosed vessels, 2:Three stenosed vessels, n.s: non‐significant, *P < .01, **P < .001, ***P < .0001

Significant higher plasma MMP‐3 and ‐9 levels were found in CAD patients with two‐ or three‐vessel disease compared to controls or patients with one vessel disease (*P* < 0.0001). TIMP‐1 and TIMP‐2 levels were lower in all patient subgroups compared to controls (*P* < 0.0001), but not statistically different between them. Apo‐CII, ‐CIII and ‐E plasma levels were significantly increased in all subgroups compared to controls (*P* < 0.0001 for Apo‐CII and ‐III, *P* < 0.001 for Apo‐E) but not statistically different between them, but for Apo‐CII slightly lower in the three‐vessel disease subgroup compared to those with one vessel disease (*P* < 0.01; Figure 2A–C).

**FIGURE 3:**
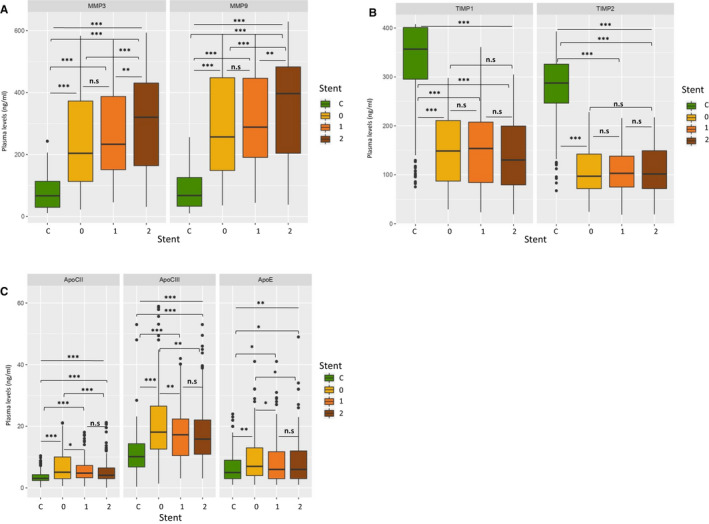
A, MMPs levels variation between different stent treatments in patient subgroups and controls. Stent treatment, C: Controls, 0: No stent treatment, 1: Bare‐metal stent, 2: Drug elution stent n.s: non‐significant, *P < .01, **P < .001, ***P < .0001. B, TIMPs levels variation between different stent treatment in patient subgroups and controls. Stent treatment, C: Controls, 0: No stent treatment, 1: Bare‐metal stent, 2: Drug elution stent n.s: non‐significant, *P < .01, **P < .001, ***P < .0001. C, Apolipoproteins levels variation between different stent treatments in patient subgroups and controls. Stent treatment, C: Controls, 0: No stent treatment, 1: Bare‐metal stent, 2: Drug elution stent, n.s: non‐significant, *P < .01, **P < .001, ***P < .0001

MMP‐3 and ‐9 plasma levels were more elevated in patients treated with stents than without (*P* < 0.0001), and ever more in patients treated with drug‐eluting stent than with bare‐metal stent (BMS) (*P* < 0.001). TIMPs levels were lowest in all subgroups but without any statistical difference according to stent treatment choices. Apo‐CII, ‐CIII and ‐E plasma levels were significantly higher in all subgroups than in controls (*P* < 0.0001), but for Apo‐E slightly in both stent treatment subgroups (*P* < 0.01), and for Apo‐CII and ‐III even higher in ‘no stent treatment’ subgroup (from *P* < 0.001 to *P* < 0.0001), and without any statistical difference between DES and BMS treatment subgroups (Figure 3A–C).

### 3.4 Heatmap and cluster analysis

**FIGURE 4:**
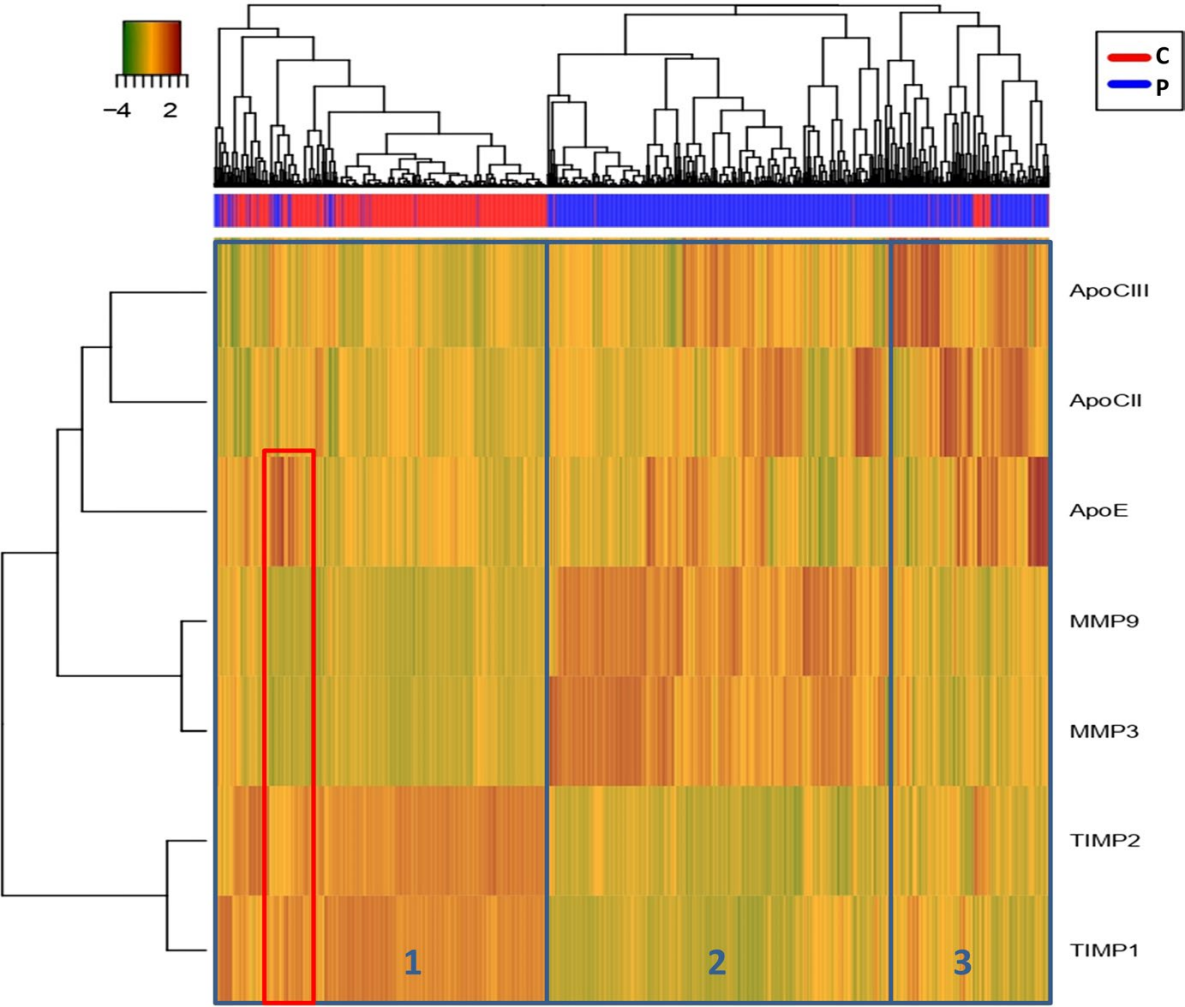
Heatmap showing study biomarkers and the associated discrimination between patient and control groups

First, we observed in variable dendogram (at left) 3 clusters matching with physiological families of dosage variable. Furthermore, MMPs and TIMPs families were more strongly correlated between them than with Apo family whereas TIMPs family was anti‐correlated with both Apo and MMPs families (All these correlations were validated using Pearson correlation’s test). For sample dendrogram (at top), we also observed rationally 3 main clusters (Pearson's dissimilarity = 0.485). Cluster 1 groups control samples but cluster 2 and 3 form a big cluster regrouping patients. Based on the heatmap, we noticed that cluster 2 represents the biggest patient gathering with higher MMP‐3 and MMP‐9 plasma levels, and lower plasma levels of TIMP‐1 and TIMP‐2. Cluster 2 also can be split into 2 sub‐clusters depending on Apo‐CII, Apo‐CIII or Apo‐E levels and the majority of these patients have normal apolipoproteins levels. Cluster 3 represents the patient group too with mostly higher levels of apolipoproteins and without elevation of MMP‐3, ‐9 and TIMP‐1, ‐2 levels, whereas control group (i.e. cluster 1) had higher TIMPs levels, lower MMPs and apolipoproteins levels. In cluster 1, we distinguish a small sub‐cluster (red framed) with higher TIMP‐1 levels, lower levels of metalloproteinases and higher Apo‐E (Figure 4).

This unsupervised classification method gives us a sensitivity (True Positive Rate) of 90.0 % with a specificity (False Positive Rate) of 8.1 %. So, this description analysis confirmed us the potential of the panel to construct a predictive model using a supervised statistical method.

### 3.5 ROC Curve analysis: panel performance study

**FIGURE 5:**
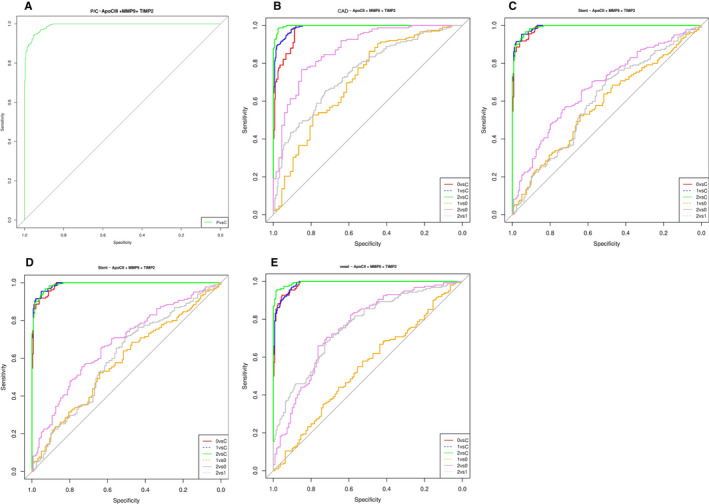
A, Apo‐CIII, MMP‐9 and TIMP‐2 in patients vs controls, AUC = 0.991. B, Apo‐CII, MMP‐9 and TIMP‐2 in CAD patients subgroups vs controls, AUC = 0.997. C, Apo‐CII, MMP‐9 and TIMP‐2 in‐stent treatment patients subgroups vs controls, AUC = 0.9915. D, Apo‐CIII, MMP‐9 and TIMP‐2 in‐stent treatment patients subgroups Vs controls, AUC = 0.9914. E, Apo‐CIII, MMP‐9 and TIMP‐2 in number of stenosed vessel vs controls, AUC = 0.995. Diagnostic performance of a multiplex protein biomarker assay (Apo‐CII or Apo‐CIII, MMP‐9 and TIMP‐2). ROC was plotted to describe performance characteristics in the 757‐subject cohort (controls and different patient's subgroups) (Figure 3A–E). AUC, area under the curve; C, control; CAD, coronary artery diseases; P, patients, CAD subgroups (0: stable angina, 1: NSTEMI, 2: STEMI), stent treatment (0: no stent treatment, 1: BMS (Bare‐metal stent), 2: DES (Drug elution stent)), number of stenosed vessels (0: one stenosed vessel, 1: two stenosed vessels, 2: three stenosed vessels)

In order to find the minimum dosage variable that classes each patient with the minimum misclassifications, we decided to test all combinations of dosage variables to find the best models. We applied the logistical regression to each combination and computed area under the curve (AUC) of receiver operator characteristic (ROC) to arrange and select the best ones. All combinations of dosage (explicative) variables represent 127 possible models that we applied to our 4 explained variables: patient/control, MI (control, stable angina, NSTEMI and STEMI), number of involved vessels (control, one vessel, two vessels and three vessels), type of stent treatment (control, no stent treatment, BMS and DES). As expected, models that combine the highest number of variables gave the best AUC values for all explained variables. We chose to focus on models with 3 variables and found that models showing the best AUC contained 3 variables coming from each of the 3 physiological groups. For patient/control, the highest AUC (0.991) was found for the “Apo‐CIII + TIMP‐2 + MMP‐9” model (Fig 5A). For CAD the highest AUC (0.998 for 2 vs C) was found for the “Apo‐CII + MMP‐9 + TIMP‐2” model (Fig. 5B). Stent revealed high AUC for both “Apo‐CII + MMP‐9 + TIMP‐2” (0.9915 for 1 vs C) and “Apo‐CIII + MMP‐9 + TIMP‐2” (0.9915 for 2 vs C) models (Fig. 5C and 5D). Regarding the number of involved vessels, the model associated with the highest AUC (0.995) was “Apo‐CIII + MMP‐9 + TIMP‐2” (Fig.5E).

## 4 DISCUSSION

The MMPs are functionally and structurally linked to the family of zinc‐dependent endopeptidases. Their biological roles have been associated with the turnover and degeneration of most of the ECM components. They are secreted zymogens (pro‐MMPs) and they are activated using other serine‐proteinases or MMPs. They have been shown to be implicated in the atherosclerotic plaque remodelling, their instability and rupture as well as thrombus formation. The interaction with glycosaminoglycans induces the specific intracellular or extracellular locations of MMPs which control their activity and specificity.^13^ MMP‐9 participates in ECM degradation and significantly regulates inflammatory response. Furthermore, MMP‐9 is responsible for fibrosis resulting in plaque instability and rupture^14^ and was shown significantly enriched in atherosclerosis plaque.^15^ The major MMPs, especially MMP‐3 and MMP‐9 which are evaluated have been implicated in plaque destabilization as well as vascular remodelling by metabolizing the collagen present in the fibrous cap.

In our study, MMP‐9, MMP‐3 blood levels are increased in CAD patients compared to healthy controls, these two biomarkers have a relevant positive correlation between them, also they are higher in the most complicated stage of CAD, that is STEMI compared to NSTEMI and stable angina and their levels change depending on the absence or presence of a stent and the use of bare‐metal or drug elution stent. In addition, we showed an increase of MMP‐3,‐9 levels in patients presenting two or three stenosed vessels compared to only one. Unusually, MMP‐9 may be useful to anticipate the stent treatment and its choice. According to previous studies, plasma MMPs levels, particularly MMP‐9, are higher in subjects with acute coronary syndromes (ACS) and contributed to the molecular process of plaque destabilization.^16^ Higher levels were shown for MMP‐3 in patients with triple vessel disease.^17^ In patients with and without ACS, an elevation of MMP‐3 was related to an increased risk of CAD.^18^ A correlation was found between MMP‐3 and all mortality causes.^19^ Similarly, a higher level of MMP‐9 was found in patients with CAD^20^, ^21^ and unstable unruptured plaque, in ruptured plaque as well as in unstable plaque than in the stable plaque in another study.^22^ Nishiguchi's^23^ study showed a higher MMP‐9 level in post stent STEMI patients, additionally in triple vessel stenosis patients.^24^ Furthermore, patients with multiple sites of in‐stent restenosis had higher MMP‐9 levels. A previous study showed an elevated MMP‐3 level in patients with arterial hypertension.^25^ However, decreased MMP‐3 levels were shown in stable and unstable CAD patients.^26^ Most of these authors suggested that MMP‐9 is a functional biomarker in CAD. A similar Tunisian study found only MMP‐3 which could be used as a cardiovascular events predictor due to mostly for their endpoint study, the smaller population study (patients/controls), patient group subdivision and the difference of cities sampling.^27^ MMP‐3 regulates vascular smooth muscle cell migration via MMP‐9 activation and the MMP‐3 expression and secretion may precede MMP‐9 expression and secretion.^28^ As shown in previous studies, MMP‐9 would be related to the stability of atherosclerotic plaques, so a higher level may indicate that plaques are vulnerable or have ruptured. We suggested that, MMP‐3, MMP‐9 was related to the CAD risk and its level might be appropriate as a clinical biomarker to predict CAD outcome.

We have established a statistically significant decrease of TIMP‐1, TIMP‐2 in CAD patients compared to controls; these inhibitors are decreased in STEMI compared to NSTEMI and stable angina and concerning the number of stenosed vessel and stent treatment, there was no statistical difference between different subgroups but there was a positive correlation between TIMP1 and ‐2. TIMP‐1 was considered as a CAD predictive biomarker.^29^ Decreased TIMP‐2 plasma levels were detected in CAD and decreased plasma levels of both TIMP‐1 and TIMP‐2 in ACS and stable angina.^30^ An association was found between TIMP‐1 and the number of stenosed vessels but not with CAD severity.^31^ Inversely to our results, TIMP‐1 was found significantly higher in triple versus single vessel disease,^32^ in the ruptured plaque group, and higher level in unstable CAD.^21^

Matrix metalloproteinases activities can be regulated by endogenous inhibitors, also called tissue inhibitors of MMPs (TIMPs), which are the major inhibitors of MMPs in tissues as well as in biological fluids. We have found a negative correlation between MMPs and TIMPs in accordance with their inhibitory effect. Some previous studies have tried to illustrate the relation between MMPs and TIMPs with various results. Although the influence of TIMPs is still uncertain on cardiovascular disease prognosis, it has been suggested that local factors could contribute to the alteration of the balance between MMPs and TIMPs thus favouring an excess of proteolysis and resulting in disease progression.^33^ Moreover, the effects on cell growth and migration, angiogenesis, apoptosis and differentiation are the most significant activities of TIMPs which are independent of MMPs.^34^ TIMPs levels are either elevated or decreased in CAD patients and several studies have shown inverse correlations between MMPs and TIMPs levels in these patients.^35^, ^36^ TIMP‐1, and mostly TIMP‐2 being overexpressed and inhibiting MMP‐9, are known as inhibitors of atherosclerotic plaque development and instability, through modulation of macrophage and foam cell behaviour, perturbing intra‐plaque MMP activity and increasing lesion stability.^36^ It was suggested that a change in MMP/TIMP balance could have an importance in hypertensive heart disease.^37^ Another author found that patients with CAD had higher MMP‐9 and TIMP‐1 plasma levels but a lower TIMP‐2 level,^38^ also both increased MMP‐9 and TIMP‐ 1 plasma levels in premature coronary atherosclerosis patients, but with lower MMP‐3 and TIMP‐2 levels.^39^ Notably, increased plasma levels of both MMP‐9 and TIMP‐1 were identified in patients with CAD,^40^ as well as in patients with angina or MI.^41^ Higher plasma MMP‐9 and TIMP‐1 levels were also found in NSTEMI.^42^ Giagtzidis's team has found that patients with peripheral arterial disease undergoing angioplasty/stenting had MMP‐3 elevation, but not for MMP‐9, TIMP‐1 and ‐2.^43^ Moreover, MMP‐9 levels were higher in ACS than in stable angina, but TIMP‐1 showed no difference.^44^ MMP‐9 level was increased in STEMI patients also in smokers and with no difference for TIMP‐1, and with no correlation between MMPs and TIMPs.^45^

Apo‐CII and Apo‐CIII were significantly increased in CAD patients compared to controls; they showed a statistically significant variation when comparing different patient subgroups and they were in positive correlation with MMPs and in negative correlation with TIMPs. It is the first study which investigates this correlation and we suggested a synergic action between MMPs and apolipoproteins in cardiovascular complications. In previous studies, Apo‐CIII and Apo‐E have been associated with a higher risk of diabetes^46^; their blood levels were increased in chronic heart disease (CHD) in relation to dyslipoproteinaemia^47^ and they were elevated in CAD.^48^ High Apo‐CIII level was found in CAD patients,^49^ as well as in patients with familial hyperlipidemia and cardiovascular disease.^50^ Apo‐CII and Apo‐E were higher in function of stenosis progression.^51^ Apo‐CII and ‐CIII are considered as endogenous mediators of lipoprotein lipase activity and affect the concentration of triglycerides in blood which leads to aggravation of aortic atherosclerosis, thereby increasing the inflammatory response.^10^ In discordance to our study, lower levels of Apo‐CII, Apo‐CIII and Apo‐E were described in CAD patients.^52^, ^53^ Moreover, they showed no significant differences in peripheral vascular disease,^54^ and a lower Apo‐CII was found in STEMI patients^55^ as well as depending on CAD severity with the number of stenosed vessels.^56^ Previous study has not confirmed or clarified the relation between MMPs and apolipoproteins; they just hypothesized that apolipoproteins are substrates of MMPs implicated in the accumulation of cholesterol in atherosclerotic lesions.^3^, ^11^, ^12^

The use of heatmap was very important in starting our study, it was used to give and lead ideas concerning the next statistical test, and the clustering helped to know the relation between biomarkers and their distribution between patients and controls, also to have the idea to do the ROC test. The ROC test was used to select the performed biomarkers panel to evaluate cardiovascular severity and to assess the importance of biomarkers panels of the severity of CAD. With ROC curve result, we found that MMP‐9 was more predictive for cardiovascular events than MMP‐3. Our findings show for the first time that a combination of biomarkers including one MMP (mostly MMP‐9), one TIMP (surely TIMP‐2) and one Apo‐C (II or III) can predict CAD aggravation. MMP‐9 level could be determined as an initial marker of severity in patients with CAD and probably a risk factor for the development of complications, in particular depending on the number of stenosed vessels, also for the choice of stent treatment. TIMP‐2 decrease during the acute stage of CAD suggests TIMP‐2 as a marker of vulnerable plaque, and it was always better than TIMP‐1. The choice of an Apo‐C is more difficult as Apo‐CII was a better marker than Apo‐CIII for the number of stenosed vessels, but inversely Apo‐CIII was better than Apo‐CII for the type of stent treatment. Apo‐E never was discriminative in function of CAD aggravation and was discarded from our ‘CAD aggravation panel’, finally combining MMP‐9, TIMP‐2 and Apo‐CIII with best AUC.

Our study is the first looking for the relation between MMPs, TIMPs and apolipoproteins; we support that this panel might be helpful to more detailed risk stratification in patients with CAD, helpful to predict cardiovascular disease and therapeutic procedure.

## 5 CONCLUSIONS

Based on our findings and supportive evidence in the literature, we explored prospectively the association between MMPs level, their imbalance with TIMPs (a reverse correlation) and clinical cardiovascular outcomes and their predictive value for CAD complications. The unbalance between MMPs and TIMPs in vascular wall creates favourable conditions for plaque disruption. The ‘MMP‐9 + TIMP‐2 + Apo‐CIII’ panel was the better test for follow up to avoid further complications. It might be a useful marker for continuous monitoring of CAD and for optimizing individual therapy. In particular, physicians could use this panel to improve medical care for CAD patients, ranging from medical treatment optimization, emergency coronary angiography, stent implantation and even the choice of stent type.

However, our study has some limitations such as the size of the studied population, their geographical localization and the follow‐up period. The descriptive clinical study is used to draw hypothesis which must obviously be confirmed in animal or cellular models.

## CONFLICT OF INTEREST

The authors whose names are enrolled in this study proclaim that there is no disagreement of interest, and declare that they have no association with or connection in any organization or entity with financial or non‐financial relevance (such as knowledge or beliefs, affiliations, professional or personal relationships) in the subject issue or materials discussed in the article. There is no conflict of interest between authors.

## AUTHOR CONTRIBUTION

**Assia Ben Braiek:** Conceptualization (lead); Data curation (lead); Formal analysis (lead); Investigation (lead); Methodology (lead); Software (equal); Supervision (lead); Validation (lead); Visualization (lead); Writing‐original draft (lead); Writing‐review & editing (lead). **Hinda Chahed :** Conceptualization (equal); Data curation (equal); Methodology (supporting); Validation (equal); Visualization (equal); Writing‐original draft (equal); Writing‐review & editing (equal). **florent dumont:** Data curation (equal); Formal analysis (equal); Methodology (equal); Software (equal); Writing‐original draft (equal). **abdelhak fodha:** Conceptualization (equal); Data curation (equal); Methodology (supporting); Validation (equal); Visualization (equal); Writing‐original draft (equal); Writing‐review & editing (equal). **Hichem Denguir:** Conceptualization (equal); Data curation (equal); Validation (equal); Writing‐review & editing (equal). **Habib Gamra:** Data curation (equal); Validation (equal). **Bruno Baudin:** Conceptualization (equal); Formal analysis (equal); Funding acquisition (lead); Investigation (equal); Methodology (equal); Project administration (lead); Resources (lead); Supervision (equal); Validation (equal); Visualization (equal); Writing‐original draft (equal); Writing‐review & editing (equal).

## Data Availability

The data that support the findings of this study are available from the corresponding author upon reasonable request.
